# Evolutionarily conserved structural changes in phosphatidylinositol 5-phosphate 4-kinase (PI5P4K) isoforms are responsible for differences in enzyme activity and localization

**DOI:** 10.1042/BJ20130488

**Published:** 2013-07-26

**Authors:** Jonathan H. Clarke, Robin F. Irvine

**Affiliations:** Department of Pharmacology, Tennis Court Road, Cambridge CB2 1PD, U.K.

**Keywords:** dimerization, lipid kinase activity, phosphatidylinositol 5-phosphate (PtdIns5*P*), phosphatidylinositol 5-phosphate 4-kinase (PI5P4K), NLS, nuclear localization sequence, PI4P5K, PtdIns4*P* 5-kinase, PI5P4K, PtdIns5*P* 4-kinase

## Abstract

Mammals have genes coding for three PI5P4Ks (PtdIns5*P* 4-kinases), and these have different cellular localizations, tissue distributions and lipid kinase activities. We describe in the present paper a detailed molecular exploration of human PI5P4Ks α, β and γ, as well as their fly and worm homologues, to understand how and why these differences came to be. The intrinsic ATPase activities of the three isoforms are very similar, and we show that differences in their G-loop regions can account for much of their wide differences in lipid kinase activity. We have also undertaken an extensive *in silico* evolutionary study of the PI5P4K family, and show experimentally that the single PI5P4K homologues from *Caenorhabditis elegans* and *Drosophila melanogaster* are as widely different in activity as the most divergent mammalian isoforms. Finally we show that the close association of PI5P4Ks α and γ is a true heterodimerization, and not a higher oligomer association of homodimers. We reveal that structural modelling is consistent with this and with the apparently random heterodimerization that we had earlier observed between PI5P4Kα and PI5P4Kβ [Wang, Bond, Letcher, Richardson, Lilley, Irvine and Clarke (2010), Biochem. J. **430**, 215–221]. Overall the molecular diversity of mammalian PI5P4Ks explains much of their properties and behaviour, but their physiological functionality remains elusive.

## INTRODUCTION

PI5P4Ks (PtdIns5*P* 4-kinases, EC 2.7.1.149) phosphorylate PtdIns5*P* to PtdIns(4,5)*P*_2_ [1]. Although this serves as another route of PtdIns(4,5)*P*_2_ synthesis, alternative to the 5-phosphorylation of PtdIns4*P* catalysed by the PI4P5Ks (PtdIns4*P* 5-kinases), the much lower cellular levels of PtdIns5*P* compared with PtdIns4*P* [2–4], plus the evidence for the major pathway of PtdIns(4,5)*P*_2_ synthesis *in vivo* being via the PI4P5K route [5,6], has led to a consensus that the most likely function of PI5P4Ks is to regulate the levels of their substrate PtdIns5*P* (for examples, see [7–10]). PtdIns5*P* has been proposed to have a number of functions in the nucleus [11,12] and cytoplasm [8,13–15]. The route of PtdIns5*P* synthesis is not certain; one possibility is by 4-dephosphorylation of PtdIns(4,5)*P*_2_ [16,17], and 3-dephosphorylation of PtdIns(3,5)*P*_2_ by myotubularins is another route that has experimental support [15,18].

Even though the PI5P4K family has significant catalytic site structural similarity to the protein kinase superfamily [19], previous studies have shown large differences between the specific activities of the three mammalian PI5P4Ks when assayed under the same experimental conditions, with PI5P4Kα being significantly more active than PI5P4Kβ [20,21], and the PI5P4Kγ isoform having little or no intrinsic PI5P4K activity [22]. Although we show below that some of these differences are accounted for by differing ATP affinities of the three isoforms, even at physiological ATP levels their PI5P4K activities are still orders of magnitudes apart, which raises some fundamental questions about how and why this came to be. One suggestion may be that the more active PI5P4Kα can be targeted to different cellular locations by its association with the less active isoforms, such that PI5P4Kβ targets it to the nucleus [20,21] and PI5P4Kγ to an as yet undefined intracellular vesicular compartment [9,22,23]. This would require the isoforms to associate with each other, as has been shown with PI5P4Kα and PI5P4Kβ *in vivo* [20,21], either as complexes of homodimers or as heterodimers. However, the enzymatic activity of PI5P4Kβ appears to have a functional role [12,24], so the question of how the sequence differences in these enzymes relate to functional activity differences and why these three enzymes are so different in their activity remain unresolved, yet central to our understanding of these enigmatic lipid kinases.

We have undertaken a detailed biochemical and evolutionary study of the three mammalian PI5P4Ks. As a protein family, the PI5P4Ks share a high sequence similarity at the amino acid level, suggesting that, although the common structural architecture defines similar mechanisms of action [19,25], the subtle functional differences between these isoforms must be encoded in the regions of disparity. We have investigated how the sequence differences in this enzyme family reflect structure, function and molecular evolution.

## EXPERIMENTAL

### PI5P4K recombinant protein expression and mutagenesis

Constructs harbouring human *PIP4K2* (HGNC approved symbol, previously *PIP5K2*) genes [22] were used to generate PCR fragments that were subcloned into the pGEX6P vector (GE Healthcare). Specifically, these were *PIP4K2A* (UniGene 138363), *PIP4K2B* (UniGene 171988) and *PIP4K2C* (UniGene 6280511). PCR fragments were also subcloned from constructs encoding the *Drosophila melanogaster PIP4K* gene (FlyBase identifier FBgn0039924; a gift from Dr Raghu Padinjat, National Centre for Biological Sciences, Tata Institute of Fundamental Research, Bangalore, India) and the *Caenorhabditis elegans PPK-2* gene (WormBase identifier WBGene00004088; a gift from Dr Wiebke Sassen, Georg-August-Universität, Drittes Physikalisches Institut, Göttingen, Germany). These clones were subjected to site-directed mutagenesis (either using sequential rounds of mutagenesis with primers generating single codon changes or using primers generating multiple codon changes) using the QuikChange® technique (Agilent Technologies) to produce chimaeric and kinase-dead versions. BL21(DE3) *Escherichia coli* clones harbouring these constructs were induced with 0.4 mM IPTG and probe-sonicated in the presence of protease inhibitors (Sigma–Aldrich P8465). GST-fusion proteins were harvested by binding to glutathione–Sepharose beads (GE Healthcare) and untagged protein was generated by *in situ* cleavage with 50 units of PreScission protease (GE Healthcare) for 4 h at 4°C. Purity was confirmed by SDS/PAGE and protein concentration was determined by colorimetric assay (Bio-Rad).

### Biochemical assays and enzyme kinetics

Lipid kinase assays were performed as described previously [20] with minor adaptations. Lipid substrate micelles were formed in kinase buffer (50 mM Tris/HCl, pH 7.4, 10 mM MgCl_2_, 80 mM KCl and 2 mM EGTA) by sonication. The substrate was presented as PtdIns5*P* micelles (6 μM), as a component of artificial plasma membrane liposomes [26] or in a hexagonal-phase phosphatidylethanolamine carrier (6 μM PtdIns5*P* and 60 μM phosphatidylethanolamine). Recombinant lipid kinase was added to the reaction mixture (200 μl final volume) with 10 μCi of [γ-^32^P]ATP for 10–30 min at 30°C. Lipids were extracted using acidic phase-separation [27] and separated by one-dimensional TLC (2.8:4:1:0.6 chloroform/methanol/water/ammonia, by vol.). Radiolabelled PtdIns(4,5)*P*_2_ spots were detected by autoradiography, extracted and counted with Ultima Gold XR scintillant (Packard) on a LS6500 scintillation counter (Beckman Coulter). For kinetic studies, standardized assays using increasing concentrations of unlabelled ATP (0–150 μM) were used to determine *K*_m_ values for ATP. Turnover number (*k*_cat_) values were calculated using Prism 5 (GraphPad) and a conversion factor was used for histidine-tagged fusion constructs (PI5P4Kβ+ and PI5P4Kγ+) using His_6_-tagged PI5P4Kα as standard.

Assays to determine intrinsic ATPase activities of the enzymes were completed using the Transcreener ADP^2^ fluorescence polarization method (BellBrook Labs). A range of enzyme concentrations was assayed with ATP substrate (100 μM ATP, 60 min of incubation at 22°C) and polarization units (mP) were read using a PHERAstar Plus microplate reader (BMG Labtech). Experimental values were interpolated from an ADP/ATP utilization standard curve and plotted using non-linear regression analysis with Prism 5.

### Dimerization studies

Mixtures of untagged purified recombinant PI5P4Kα and PI5P4Kγ were allowed to reach equilibrium for 16 h at 4°C in PBS. Proteins were then cross-linked by incubation with a 50-fold molar excess of BS^3^ [bis(sulfosuccinimidyl)suberate] for 30 min and the reaction was quenched by the addition of 50 mM Tris/HCl, pH 7.4, for 15 min, both at 22°C. Proteins were then thermally denatured to prevent any further potential dimerization in the assay. Samples of these mixtures were run on SDS/PAGE (8% gels) with recombinant protein controls and Western blotted with antibodies raised against PI5P4Kα (rat monoclonal) and PI5P4Kγ (rabbit polyclonal) as described previously [22]. Parallel samples, without higher multimeric or aggregated forms of the proteins (as assayed this way), were analysed by sandwich ELISA. Anti-PI5P4Kα antibody was coated on to 96-well polysorp plates (Nunc-Immuno) for 16 h at 4°C, washed with PBS and blocked with 2% BSA for 2 h. Protein complex was added to the wells (in PBS plus 0.2% BSA) for 2 h at room temperature and washed off. Captured protein was detected with anti-PI5P4Kγ antibody and horseradish peroxidase-conjugated anti-rabbit IgG secondary antibody (Pierce), each for 1 h at room temperature. After incubation with TMB (3,3′,5,5′-tetramethylbenzidine) substrate for 30 min, reactions were stopped with 30% H_2_SO_4_ and the chromogenic change was measured at 450 nm on a Multiskan Ascent plate reader (Labsystems).

### Sequence comparison and structural modelling

DNA and protein alignments were made using DNADynamo (Blue Tractor Software). Crystal structure data for PI5P4Kα (PDB code 2YBX), PI5P4Kβ (PDB code 1BO1 [19]) and PI5P4Kγ (PDB code 2GK9) were used to formulate predictive full models of the three isoforms using the UCSF Chimera package [30]. Disordered regions were confirmed as loops without any predicted sheet or helix secondary structure using GOR V [31]. Loop optimization was performed using Modeller [32] to produce ten energetically stable loop conformations for each disordered region, of which those with the best modeller objective function values were assessed with PDBsum, including PROCHECK (EMBL-EBI). The highest-quality models were selected as predicted structures within these theoretical confines. Modelled ribbon structures of each isoform were superimposed using Matchmaker [33] and regions of variability in the structural alignments were highlighted for experimental analysis.

### Evolutionary analysis

Annotated sequences for PI5P4K isoforms were obtained from all vertebrate genomic databases by gene-name and orthologue searches (Ensembl). Phylograms were obtained from the EnsemblCompara GeneTrees resource [34] to predict gene orthologies and paralogies as maximum likelihood phylogenetic gene trees (generated by TreeBeST). Newick format tree files were converted into Nexus format files [35] and the software package r8s [36] was used to estimate divergence times from branch length values of relative substitution frequencies. The resulting tree graphics were generated using TreeGraph 2 [37]. Graphical representations of multiple sequence alignments producing consensus sequences were generated using WebLogo 3 [38].

## RESULTS

### Biochemical analyses indicate different activities of PI5P4K isoforms

We have previously reported that, using specific activities under the same assay conditions [20–22], the three PI5P4K isoforms differ widely in activity, with PI5P4Kα being the most active and PI5P4Kγ being the least active by several orders of magnitude. Enzymatic assays using PI5P4Ks overexpressed in mammalian cells showed no change in activity when the substrate was presented as artificial membranes or as a hexagonal-phase lipid, and no other inositol lipid we tested acted as a better substrate than PtdIns5*P* (results not shown). It remains a possibility that a post-translational modification in eukaryotic cells has a major effect on one or more of the isoforms to make their activity levels more similar, but as precipitation of any endogenous or transfected PI5P4K from cell lines unavoidably results in co-precipitation of another isoform(s) because of their heterodimerization ([20,21] and see also below), we cannot address this issue directly.

To obtain further quantitative insight into how isoform structure differentially affects activity, we undertook a more detailed study of the recombinant proteins to compare turnover rates for each isoform based on *K*_m_ values for ATP (Table 1 and Supplementary Figure S1 at http://www.biochemj.org/bj/454/bj4540049add.htm). It is apparent that some of the differences in specific activity that we reported previously [20,22] are due to differences in ATP affinities, but the data in Table 1 show that the three isoforms also differ greatly in their turnover rates, such that even under physiological conditions (ATP concentration in the millimolar range, and thus saturating) they would still show very different PI5P4K activities: PI5P4Kα is 100-fold more active than PIP5Kβ and 2000-fold more active than PI5P4Kγ. In comparison with other human kinases PI5P4Kα has an intermediate turnover number, whereas PI5P4Kγ is at the lowest end of the scale (Supplementary Table S1 at http://www.biochemj.org/bj/454/bj4540049add.htm).

**Table 1:** Relative activities of wild-type and mutant PI5P4K isoforms The *K*_m_ value for ATP was determined experimentally and turnover numbers were interpolated as described in the Experimental section. PI5P4Kβ+ and γ+ clones refer to mutants with PI5P4Kα-like G-loop regions.

Isoform	*K*_m_ (μM ATP)	*k*_cat_ (s^−1^)
PI5P4Kα	3.94	1.05×10^−2^
PI5P4Kβ	67.05	9.55×10^−5^
PI5P4Kγ	94.18	5.72×10^−6^
PI5P4Kβ+ (mutant)	7.58	7.27×10^−4^
PI5P4Kγ+ (mutant)	3.96	2.57×10^−3^

We were able to estimate broadly the actual rate of PI5P4Kα activity in a cell. Data from previous DT40 cell studies give an accurate titre of cellular PI5P4Kα concentration quantified by quantitative MS as 1.5 pmol/mg of protein [20]. Assuming an average DT40 cell size of 10 μm diameter, this number can be converted into a *V*_max_ of approximately 1×10^3^ PtdIns5*P* molecules phosphorylated per s per cell, although this could be higher depending on the PtdIns5*P* concentration local to the enzyme.

### PI5P4Kγ is a fully functional kinase

**Figure 1:**
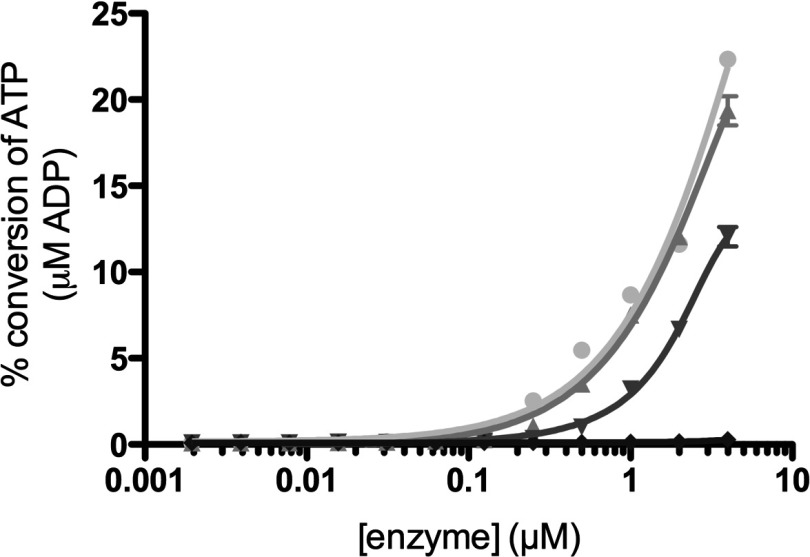
Intrinsic ATPase activities of PI5P4K isoforms The percentage of ATP consumed over a range of enzyme concentrations was plotted for each PI5P4K isoform and compared with the kinase-dead control (●, PI5P4Kα; ▲, PI5P4Kβ; ▼, PI5P4Kγ; ◆, PI5P4Kγ kinase-dead). ATP concentration in the assay was 100 μM. Result are means of triplicate samples at each enzyme concentration and error bars indicate±S.E.M.

To investigate whether the comparatively very low activity of the PI5P4Kγ isoform is due to the fact that it has a reduced ability (compared with the other two isoforms) to interact with or phosphorylate PtdIns5*P*, or whether it is actually a disabled kinase enzyme (i.e. poor in binding to or utilizing ATP), the intrinsic ATPase activity of all three isoforms was measured (Figure 1). This assay monitors the ATP turnover in the absence of lipid substrate as futile cycling, a property exhibited by most kinases (for examples see [39,40]). The data in Figure 1 show that in comparison with a kinase-dead PI5P4Kγ mutant (D280K) control, all three isoforms showed low levels of intrinsic ATPase activity, as would be expected from catalytically active enzymes [41]. Interestingly, in this assay PI5P4Kα and PI5P4Kβ were indistinguishable, and PI5P4Kγ is only 3–4-fold less effective than those two isoforms. A simplistic interpretation of this result is that much of the difference between the isoforms in their PI5P4K activity lies in their ability to recognize and then phosphorylate their lipid substrate.

### Activity of PI5P4Kγ and PI5P4Kβ can be altered by mutation

**Figure 2:**
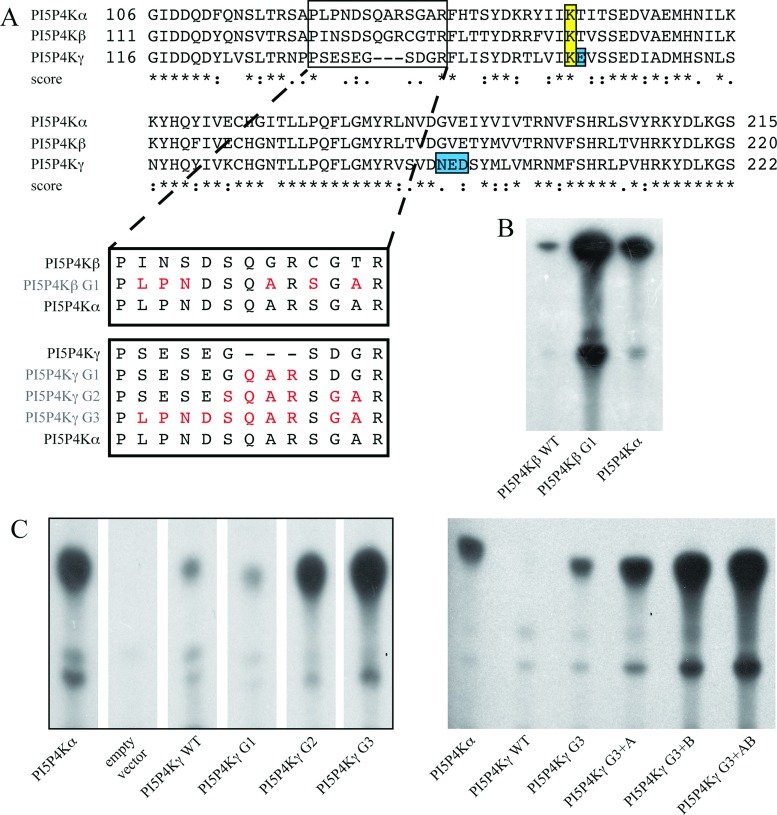
Mutation of PI5P4Kβ and PI5P4Kγ sequences increases lipid kinase activity (**A**) Alignment of fragments of the three PI5P4K amino acid sequences in the region containing the putative G-loop (boxed). Linked boxes show the sequential mutation of the wild-type sequences to match the PI5P4Kα sequence, with the mutant constructs labelled in red. The conserved catalytic lysine residue (boxed, yellow) and residues additionally mutated in PIP4Kγ (boxed, blue) are shown. Scores represent matches; *, identical; :, conserved;., semi-conserved. (**B**) Example of lipid kinase activity of the resulting mutant PI5P4Kβ protein (15 μg) compared with wild-type. (**C**) Left-hand panel: example of lipid kinase activity of the resulting mutant PI5P4Kγ proteins (200 μg) compared with wild-type (G1–G3). Lanes are reordered from the same TLC plate. Right-hand panel: additional mutants were made using the PI5P4Kγ G3 construct (A=E156T, B=N198G+E199V+D200E) and showed further increases in activity (20 μg per sample). Positive control was 50 ng of PIP4Kα, negative control was 200 μg of purified protein expressed from the empty pET vector. PIP4Kα: HGNC, 8997; UniProtKB, P48426. PIP4Kβ: HGNC, 8998; UniProtKB, P78356. PIP4Kγ: HGNC, 23786; UniProtKB, Q8TBX8.

The three human isoforms of PI5P4K show a high degree of homology [9,42], with three major domains of sequence variability being the activation loop, the variable loop (where the PI5P4Kβ nuclear localization motif lies [43]), and the putative G-loop structure (Supplementary Table S2 at http://www.biochemj.org/bj/454/bj4540049add.htm), which in PI5P4Kβ has been suggested to be responsible for positioning key residues involved with stabilizing ATP in the catalytic site (Supplementary Figure S2 at http://www.biochemj.org/bj/454/bj4540049add.htm). Figure 2(A) shows the sequence alignment of the three PI5P4Ks around this G-loop region, emphasizing the clear differences between the three isoforms. To explore the significance of these, we undertook a systematic sequential mutation of the PI5P4Kβ and PI5P4Kγ putative G-loop regions to change them to the PI5P4Kα sequence (Figure 2A). These changes significantly increased the activity of these isoforms (Figures 2B–2C). Additional mutation of some residues where the PI5P4Kα and PI5P4Kβ isoforms are identical and different from PI5P4Kγ, and which are likely to interact with the PtdIns5*P* substrate ([19] and Figure 2A), further enhanced activity (Figure 2C). These mutations included a key amino acid close to one of the conserved catalytic residues in PI5P4Kγ (see [19]) plus three amino acids in the vicinity of the potential gatekeeper residue [44].

**Figure 3:**
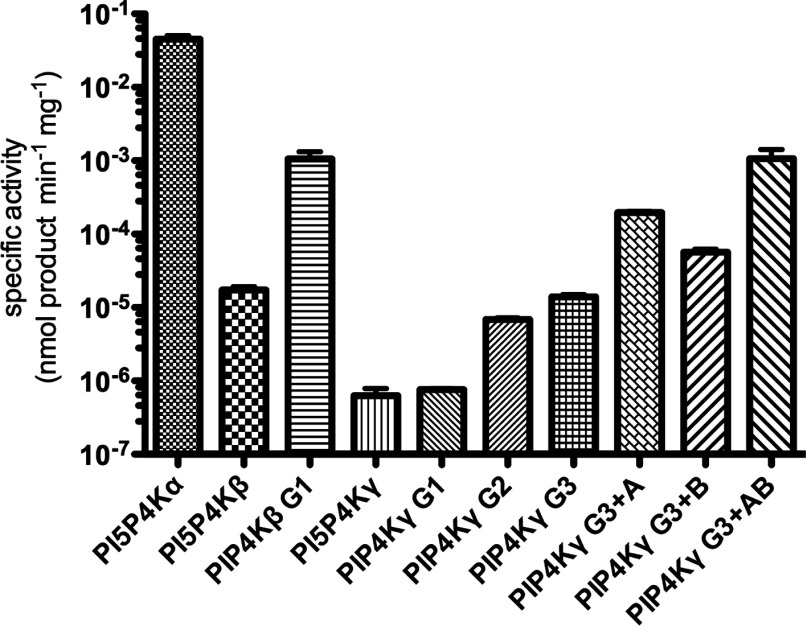
Specific activities of all mutant PI5P4Kβ and PI5P4Kγ constructs In comparison with wild-type PI5P4Kα control (50 ng), PI5P4Kβ mutant G1 showed enhanced activity over wild-type PI5P4Kβ (15 μg). Similarly, mutation of the PI5P4Kγ G-loop (G1–G3) enhanced activity over wild-type PI5P4Kγ (200 μg). Additional mutations further increased the activity of mutant PI5P4Kγ G3 (A, B and A+B, 20 μg of each). At least four replicates of each sample were used (*n*=15, 7, 7, 10, 4, 4, 4, 6, 6 and 9 respectively from left to right). For details of specific mutants see Figure 2.

A comparison of the specific activities of all of the mutants is shown in Figure 3 (note the logarithmic scale). The most active mutants were designated as PI5P4Kβ+ (PI5P4Kβ G1, Figure 3) and PI5P4Kγ+ (PI5P4Kγ G3+AB, Figure 3) and enzyme turnover rates were quantified and calculated for these mutants for comparison with the wild-type enzymes (Table 1 and Supplementary Figure S1). Although the most active PI5P4Kβ and PI5P4Kγ mutants are not as active as PI5P4Kα (Figure 3), it is apparent that they are approaching that activity, and thus that, certainly for PI5P4Kγ, we have countered and thus explained much of the low PI5P4K activity of this isoform.

### Heterodimerization between PI5P4K isoforms

The dimerization interface along the PI5P4K β1-sheet domain has been predicted to allow hetero- as well as homo-dimerization [19], and apparent heterodimerization has been shown to occur in cells between PI5P4Kα and PI5P4Kβ [20,21], probably at random, governed only by the relative abundance of the two isoforms [20]. Activity measurements suggested indirectly that heterodimerization with PI5P4Kβ may alter the enzymatic activity of PI5P4Kα [21].

**Figure 4:**
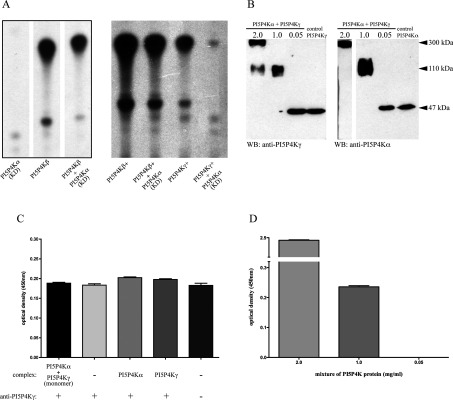
Heterodimerization of PI5P4Ks (**A**) An example of activity assays using purified recombinant PI5P4Ks showing reduced PtdIns(4,5)*P*_2_ production with 100 μg of kinase-dead PI5P4Kα (KD), PI5P4Kβ or a combination (200 μg) of the two (left-hand panel, lanes reordered from the same TLC plate) or 10 μg of active mutants of PI5P4Kβ and PI5P4Kγ (+) in combination with 10 μg of kinase-dead PI5P4Kα (right-hand panel). (**B**) Western blots of mixtures of PI5P4Kα and PI5P4Kγ (after cross-linking) at different protein concentrations promoting formation of monomers, dimers and higher oligomeric forms. The same mixtures were blotted with antibodies against each PI5P4K, using the relevant purified isoform as a control (not cross-linked). The 2.0 mg/ml lane on the PI5P4Kα blot is a longer exposure of the same blot. (**C**) Sandwich ELISA controls were not significantly different (ANOVA using Bonferroni's multiple comparison test) to signal obtained detecting PI5P4Kγ in a cross-linked monomer dilution. Controls indicated no signal above background (no complex, no antibody) for the detection blank or cross-reactivity of the antibodies. (**D**) Strong positive signal was detected from the assay using complex at multimer concentration as a positive control. Significant capture of PI5P4Kγ signal was also detected in a protein mixture comprising dimers (ANOVA using Tukey's multiple comparison test), against the monomer dilution (values background subtracted). *n*=8 for all samples, results are means±S.E.M.

Activity assays using mixtures of different PI5P4K isoforms with kinase-dead PI5P4Kα show a reduction in activity in all cases, suggesting that there may be a combinatorial enhancement to activity within the dimer (Figure 4A), but this may also be explained as an inhibition of activity caused by association of different homodimers to form tetramers, an association that has been shown to occur in the crystal structure of PI5P4Kγ (PDB code 2GK9) and *in vitro* (below). Indeed, the association of PI5P4Kα with PI5P4Kβ in cells [20,21] and of PI5P4Kα with PI5P4Kγ *in vitro* [22] could all be explained by interaction of homodimers to form heterotetramers (or higher multimeric states), and true heterodimerization has not been rigorously established for any PI5P4Ks.

Predictive full models of the three isoforms were created *in silico* using existing structural data, and estimated conformations were modelled for the disordered regions and assessed for viability as described in the Experimental section. Predicted ribbon structures were aligned along the dimerization plane and are presented as homodimers (Supplementary Figure S3 at http://www.biochemj.org/bj/454/bj4540049add.htm), and further to previously published work regarding the energetic feasibility of PI5P4Kα–PI5P4Kβ heterodimers [20], our modelling also suggests that the dimerization interface would support PI5P4Kα–PI5P4Kγ and PI5P4Kβ–PI5P4Kγ heterodimers (Supplementary Figure S3), with predicted number and lengths of potential hydrogen bonds similar to homodimer structures (Table 2).

**Table 2:** Comparison of dimerization interfaces for PI5P4K homo- and hetero-dimers Frequency and length of hydrogen bonds from available crystal structures and potential bond numbers and lengths from modelled heterodimers as described in the Experimental section. 1 Å=0.1 nm.

PI5P4K dimer (monomer–monomer)	Number of hydrogen bonds	Average bond length (Å)
α–α	11	2.93
β–β	7	2.97
γ–γ	9	2.90
α–β (modelled)	9	3.00
α–γ (modelled)	7	3.22
β–γ (modelled)	8	2.65

To address this issue experimentally we used purified recombinant PI5P4Kα and PI5P4Kγ that were un-tagged, to ensure that no interaction of tags occurred to complicate the interpretation. The two isoforms were allowed to mix at various concentrations before cross-linking the proteins (through multiple lysine residues at the dimerization interface region). Western blotting of these mixtures then indicated the different concentrations at which the proteins formed monomers, dimers or multimeric complexes (Figure 4). When these mixtures were in parallel subjected to a sandwich ELISA to capture (using anti-PI5P4Kα antibody) and subsequently detect any PI5P4Kγ isoform, a signal was observed using the concentration of protein mixture that consisted solely of dimers (Figures 4B and 4D). Controls were included to detect the level of background signal obtained from the assay, and no cross-reactivity was seen using the specific antibodies for capture or detection. The signal obtained capturing protein from the mixture conditions producing only monomers was not significantly higher than background (Figure 4C), and the protein mixture producing higher multimeric forms gave an enhanced positive signal as expected (Figure 4D), thus providing respective internal negative and positive controls. Overall the data in Figure 4 provide experimental evidence for true heterodimerization between PI5P4Kα and PI5P4Kγ, and our structural modelling described above leads us to conclude that the apparently random association between PI5P4Kα and PI5P4Kβ [20] is also a heterodimerization.

### Divergence of isoforms suggests molecular evolution

**Figure 5:**
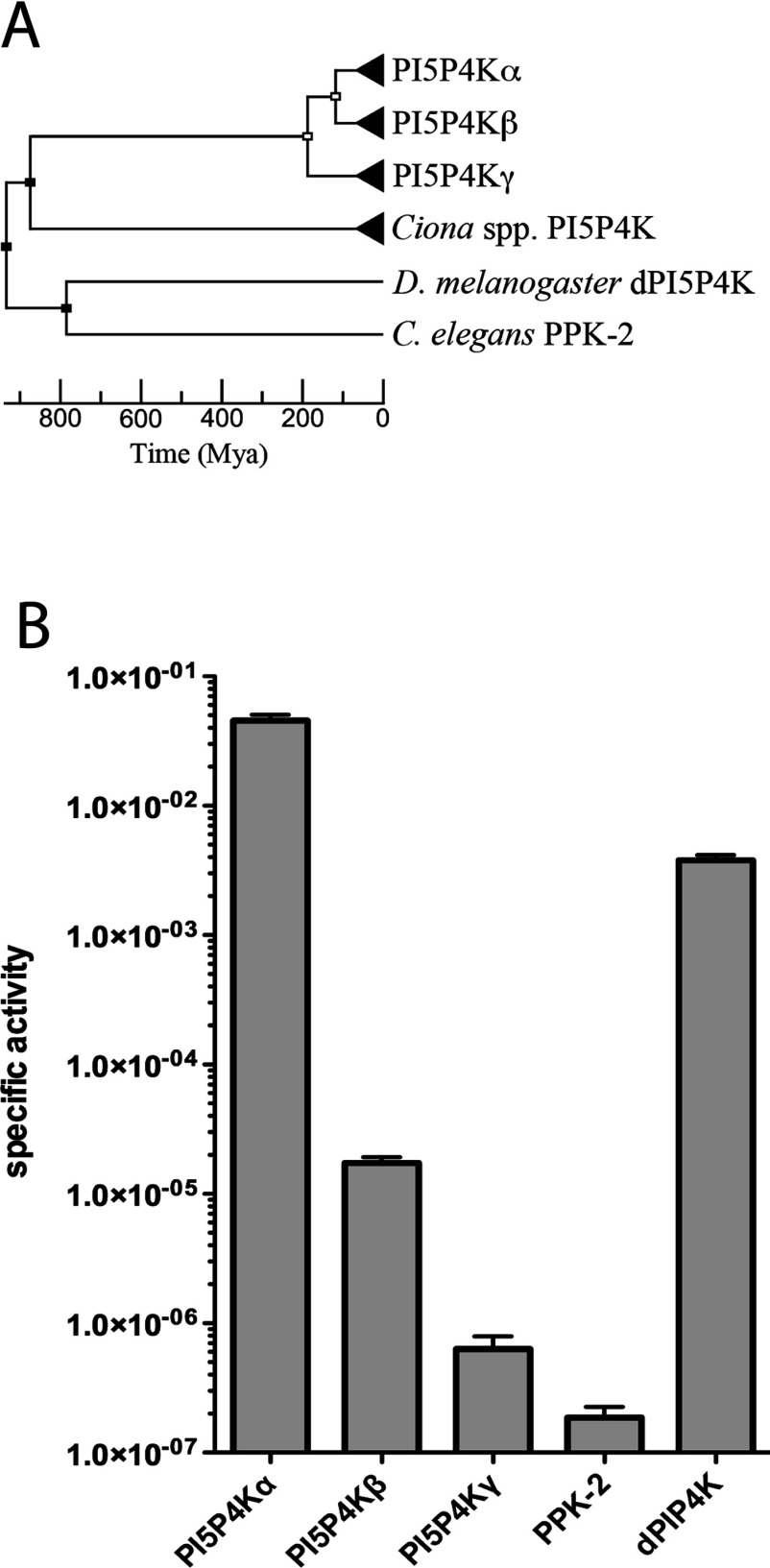
Molecular evolution of PI5P4K family activity (**A**) Bifurcating unrooted chronogram depicting the evolution of PI5P4K isoforms estimated from substitution rate branch lengths as detailed in the Experimental section. Nodes represent postulated speciation (black) or gene duplication (white) events and triangular terminal nodes represent clades. (**B**) A comparison of specific activities of human PI5P4Kα, PI5P4Kβ and PI5P4Kγ isoforms with the single *C. elegans* PI5P4K activity, PPK-2 (UniProtKB Q9BL73), and the single *D. melanogaster* activity dPIP4K (UniProtKB Q8SXX1). Activity units are nmoles of product/min per mg of enzyme. *n*=5 and results are means±S.E.M.

Screening specifically for PI5P4K enzymes by sequence similarity, the divergence of superfamily PI4P5K and PI5P4K activities seems to date back to the evolutionary appearance of the metazoans (Supplementary Figure S4 and Table S3 at http://www.biochemj.org/bj/454/bj4540049add.htm). Within the PI5P4K family a single enzyme seems to be present in invertebrate species (Figure 5A), and further genome searching using the sequence for the *D. melanogaster* gene suggested that there is also evidence for a PI5P4K activity in most, but not all, metazoan genomes so far sequenced (Supplementary Table S3). A comparison of all of the currently annotated vertebrate genomes available showed that single genes coding for the PI5P4Kα and PI5P4Kβ isoforms are conserved in every species, with the exception of duplicate copies of the PI5P4Kα gene in three fish genomes and the absence of an annotated PI5P4Kβ gene from three different species (Supplementary Table S4 at http://www.biochemj.org/bj/454/bj4540049add.htm). In contrast, the gene coding for the PI5P4Kγ isoform is present in most mammalian genomes, but conspicuously absent from any bird genomes, and is present in multiple copies in all fish genomes so far sequenced (Supplementary Table S4).

Of the three PI5P4K isoforms, the earliest divergence is apparently that of PI5P4Kγ resulting from a gene duplication event with a later divergence of PI5P4Kα and PI5P4Kβ by a second gene duplication, suggesting that these two isoforms have a closer evolutionary relationship (Figure 5A). It is therefore a crucial question whether the much lower lipid kinase activity of the PI5P4Kγ isoform was due to a subsequent loss of function, or whether the high lipid kinase activity of PI5P4Kα was caused by a gain of function (neofunctionalization). To answer this, an experimental comparison was made of the PI5P4Ks from two of the organisms expressing a single kinase isoform. We assayed the PI5P4K activity of recombinant dPIP4K from *D. melanogaster* and PPK-2 from *C. elegans*. The specific activity of dPIP4K was comparable with that of human PI5P4Kα, implying a loss of activity of PI5P4Kγ since divergence. However, PPK-2 from *C. elegans* had a similar specific activity to that of PI5P4Kγ, suggesting the exact opposite: a gain of activity by PI5P4Kα (Figure 5B: again, note the log scale). A caveat to these observations, and a probable explanation of the paradox, would be that both have continued to evolve gain or loss of function since divergence, and without accurate prediction of the ancestral sequence either outcome is possible. Put another way, we can only experimentally examine these organisms now, not millions of years ago.

**Figure 6:**
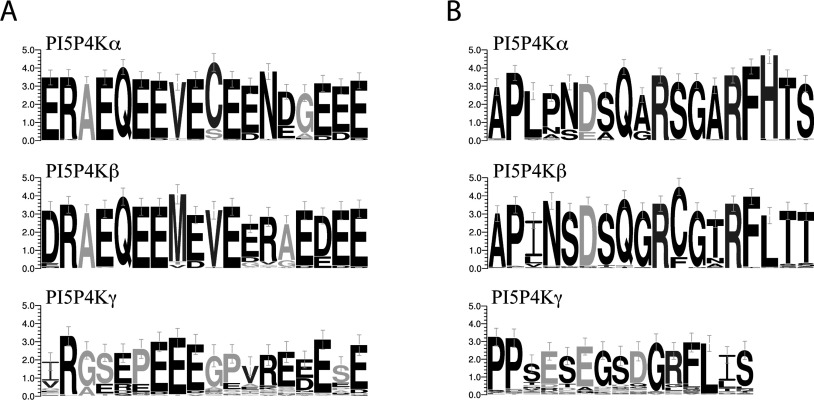
Highly conserved consensus sequences in PI5P4K isoforms (**A**) Conservation of protein sequence in the NLS region of different PI5P4K isoforms. Comparison was made of all sequences available on the Ensembl database with sequence in this region (PI5P4Kα, 53 species; PI5P4Kβ, 44 species; PI5P4Kγ, 39 species). Greyscale denotes hydrophobicity (black, hydrophilic; light grey, neutral; dark grey, hydrophobic). (**B**) Comparison of protein sequence in the G-loop region of different PI5P4K isoforms (PI5P4Kα, 52 species; PI5P4Kβ, 51 species; PI5P4Kγ, 47 species). Greyscale denotes amino acid charge (black, uncharged; dark grey, positive; light grey, negative). Units are bits and error bars represent an approximate Bayesian 95% confidence interval.

Within vertebrates, evolutionary trees were constructed for each isoform on the basis of sequence similarity, which suggested similar routes of evolution (Supplementary Figure S5 at http://www.biochemj.org/bj/454/bj4540049add.htm), although consensus of key sequences such as the NLS (nuclear localization sequence) and G-loop regions across all organisms for each isoform suggested a conserved function unique to each (Figure 6). In particular, the high degree of conservation in the PI5P4Kβ NLS resulted in strong secondary structure features, presumably conserving its nuclear localization as discussed below. Interestingly, both of the single PI5P4K enzymes in *Drosophila* and *C. elegans* show closer similarity to the PI5P4Kα consensus sequence.

### Nuclear localization of PI5P4Kβ

**Figure 7:**
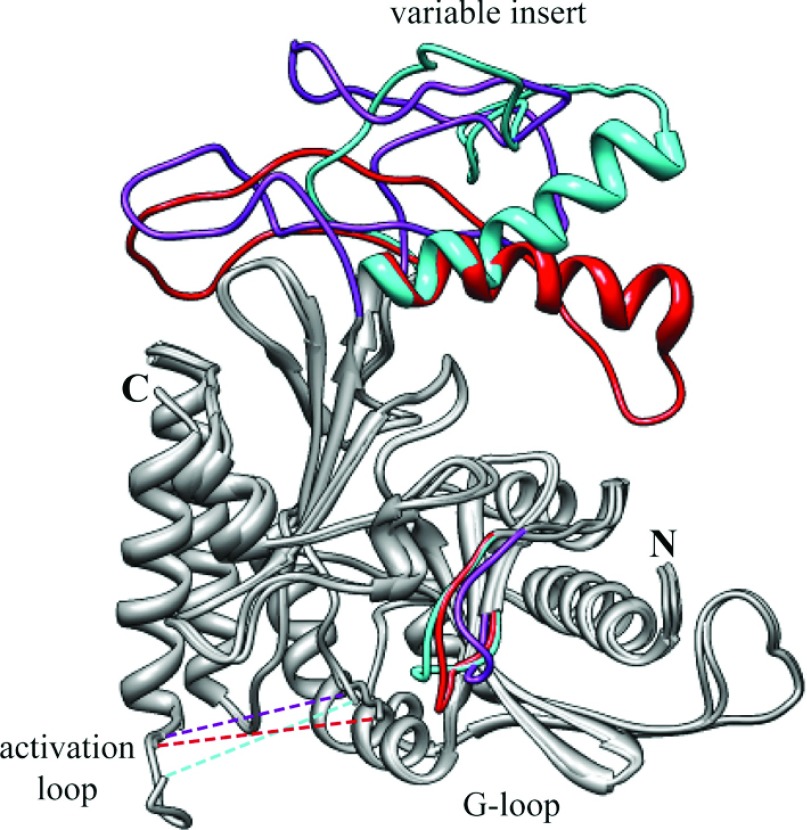
Comparison of modelled PI5P4K structures A ribbon diagram overlay of modelled structures shows close similarity (grey regions) of all three isoforms with regions of variability coloured [PI5P4Kα (PDB code 2YBX), red; PI5P4Kβ (PDB code 1BO1), cyan; PI5P4Kγ (PDB code 2GK9), purple]. The modelled structure highlights differences in the variable insert and G-loop regions (labelled). Broken lines represent the activation loop regions that were not modelled (PI5P4Kα, amino acids 366–386; PI5P4Kβ, amino acids 372–391; and PI5P4Kγ, amino acids 377–404). N-terminal peptide sequences with no crystallographic structure were omitted (PI5P4Kα, amino acids 1–33; PI5P4Kβ, amino acids 1–38; and PI5P4Kγ, amino acids 1–43). Protein N- and C-termini are labelled.

Another finding of interest emerging from our structural modelling concerns the NLS of PI5P4Kβ, which lies at the start of the variable loop [43]. Our earlier more superficial prediction was that the PI5P4Kα sequence would not be helical in this region [43], but secondary structure prediction using two separate algorithms indicates that, although the corresponding PI5P4Kγ sequence would not be helical (Supplementary Figure S6 at http://www.biochemj.org/bj/454/bj4540049add.htm), the PI5P4Kβ and PI5P4Kα NLSs would form α-helix structures. However, the structure of PI5P4Kα (PDB code 2YBX) taken together with our modelling of these regions in the present study suggests that there is an increased accessibility of this helix in PI5P4Kβ (Figure 7), which must account for the different (nuclear) location of that isoform. This is consistent with our earlier experimental observation that even a change in orientation of the relevant α-helix can alter the cellular localization of PI5P4Kβ [43].

## DISCUSSION

The clearest conclusion that can be drawn from the results of the present study is that the three mammalian PI5P4K isoforms have evolved to have very different PI5P4K activities, and that these differences are inherent within their structures. Similar intrinsic ATPase activities of the three PI5P4Ks suggests that the observed isoform-specific differences in lipid turnover are due to structural adaptations, as higher activity can be regained by mutation of amino acids to mimic the PI5P4Kα active site. The physiological reason for this difference in catalytic activity is still elusive. Our studies have not identified alternative substrates or mechanisms of substrate presentation to suggest that *in vivo* the differences may not be significant. A possibility that remains for us to explore experimentally is that one or more of these enzymes might have a physiologically significant protein kinase activity, either against themselves or each other to regulate activity, or against entirely different protein substrates, which have precedents in lipid kinases such as PI3Kγ (phosphoinositide 3-kinase γ) [45] and PI4P5Kγ [46]. It is worth noting in this context that above we calculated an approximate rate of PtdIns5*P* phosphorylation by PI5P4Kα in a DT40 cell. Were PI5P4Kγ to be expressed at a level similar to that of PI5P4Kα, it would only be metabolizing PtdIns5*P* at approximately less than 1 molecule/cell per s, which, taken together with an estimate of approximately 500000 PtdIns5*P* molecules per cell (from e.g. [3]) could be argued to support the idea of PI4P5Kγ having a natural substrate other than that lipid.

Alternatively, a primary function of the two less active isoforms may be to target the active PI5P4Kα to different locations [20,21], as their different localization sequences seem also to be inherent in their structure, at least as far as PI5P4Kβ is concerned (above). Additionally, we have also shown in the present study that, at least *in vitro*, the different PI5P4K isoforms can naturally heterodimerize, suggesting that this interaction, rather than the association of homodimers, is a viable mechanism.

There is, however, good evidence to show that the intermediate enzymatic activity of PI5P4Kβ does have physiological relevance [12,24], suggesting multiple roles for the PI5P4Ks that are possibly tissue- and cell-type-specific. Investigation of the PI5P4Ks in organisms with a single enzyme activity (*C. elegans* and *D. melanogaster*) has provided contradictory evidence to the question of whether the ancestral sequence had high or low intrinsic activity, and this opens the question as to whether a functionally active PI5P4K is required or whether organisms such as *C. elegans* have since evolved adaptive functions for this enzyme, given that the time needed for fixation of a null mutation and pseudogenization has been estimated to be short [47,48] compared with the earliest divergence of these isoforms approximately 200000000 years ago. One possibility would be that PI5P4Kγ has been retained in mammals by subfunctionalization as a protein kinase as discussed above. Further detailed investigation will be required to find answers to these new questions.

In conclusion, we believe our data throw valuable light on the role(s) of the PI5P4K family in the animal kingdom, and how their function is related to the molecular evolution that is apparent in structural differences between very similar isoforms. Addressing these questions at the physiologically functional level requires further exploration.

## Online data

Supplementary data
