# Supplementary material for: Evolutionarily conserved structural changes in phosphatidylinositol 5-phosphate 4-kinase (PI5P4K) isoforms are responsible for differences in enzyme activity and localization
Source: Biochem J. 2013 Jul 26;454(Pt 1):49–57. doi: 10.1042/BJ20130488 (PMC3749867; doi:10.1042/BJ20130488)
Supplement: Supplementary data [file bj4540049add.pdf]

BJ Signal

Biochem. J. (2013) **454**, 49–57 (Printed in Great Britain) doi:10.1042/BJ20130488

online data

SUPPLEMENTARY ONLINE DATA

Evolutionarily conserved structural changes in phosphatidylinositol 5-phosphate 4-kinase (PI5P4K) isoforms are responsible for differences in enzyme activity and localization

Jonathan H. CLARKE[1] and Robin F. IRVINE

Department of Pharmacology, Tennis Court Road, Cambridge CB2 1PD, U.K.

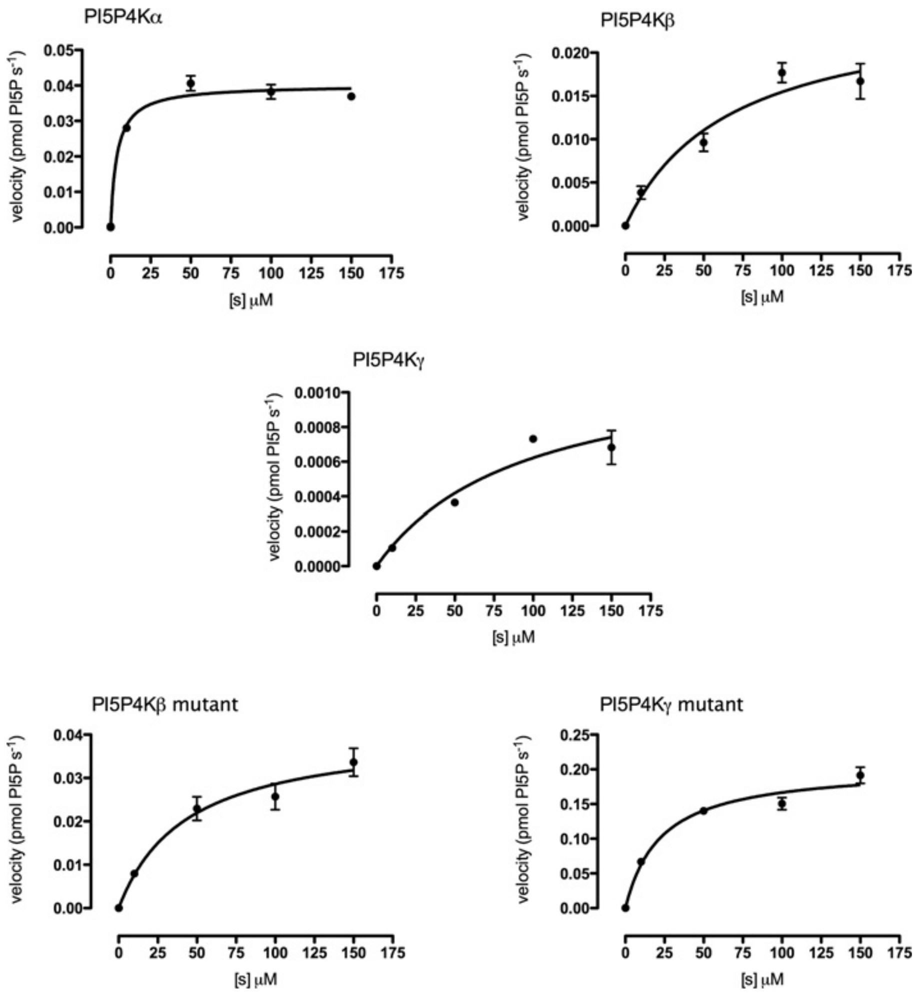

Figure S1 Saturation curves for PI5P4K enzyme activity assays at different ATP substrate concentrations

Experiments were conducted with a minimum of triplicate samples. Mutant PI5P4Kβ and PI5P4Kγ enzymes refer to constructs mutagenized to increase the intrinsic activity of the isoform (see the Experimental section and Figure 2 of the main text). Results are means $\pm$ S.E.M.

[1] To whom correspondence should be addressed (email jhc30@cam.ac.uk).

© 2013 The Author(s)
The author(s) has paid for this article to be freely available under the terms of the Creative Commons Attribution Licence (CC-BY) (http://creativecommons.org/licenses/by/3.0/) which permits unrestricted use, distribution and reproduction in any medium, provided the original work is properly cited.

Biochemical Journal www.biochemj.org

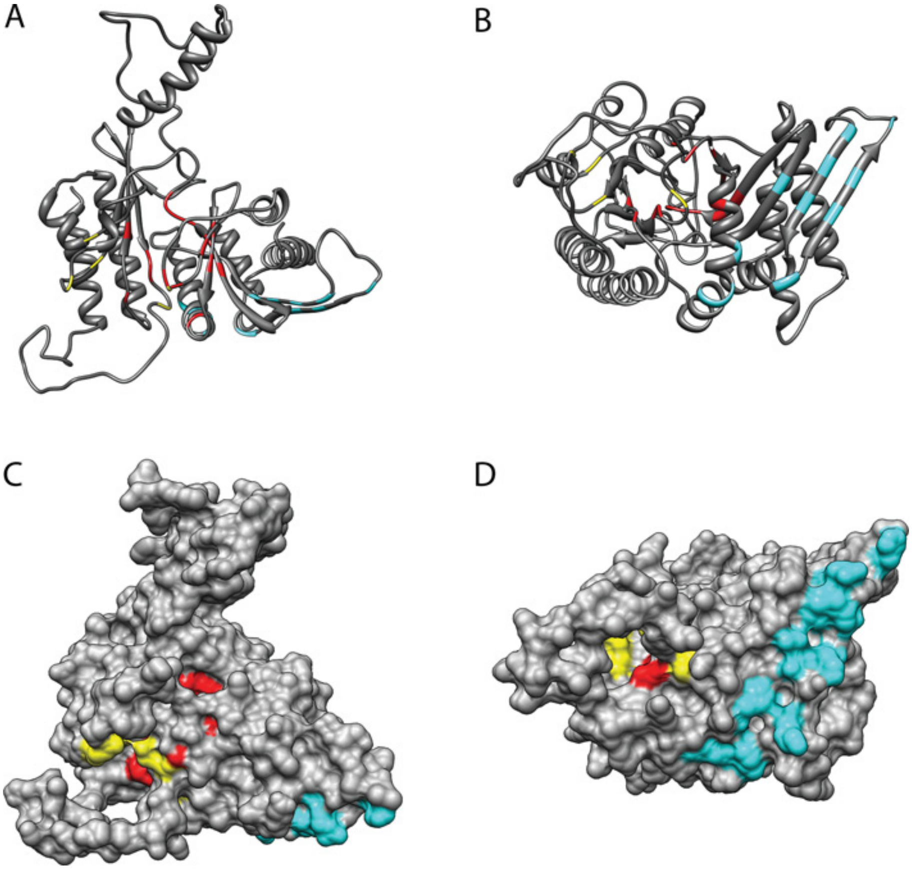

Figure S2 Models of PI5P4Kβ indicating key structural amino acids

Ribbon (**A** and **B**) and space-filling (**C** and **D**) models of PI5P4Kβ with amino acid residues suggested to be important in ATP binding (red), PtdIns5*P* binding (yellow) and membrane interaction (cyan). Side views (**A** and **C**) and membrane-binding views (**B** and **D**) are shown. Data were extracted from [1]. Models were compiled using the UCSF Chimera package.

© 2013 The Author(s)
The author(s) has paid for this article to be freely available under the terms of the Creative Commons Attribution Licence (CC-BY) (http://creativecommons.org/licenses/by/3.0/) which permits unrestricted use, distribution and reproduction in any medium, provided the original work is properly cited.

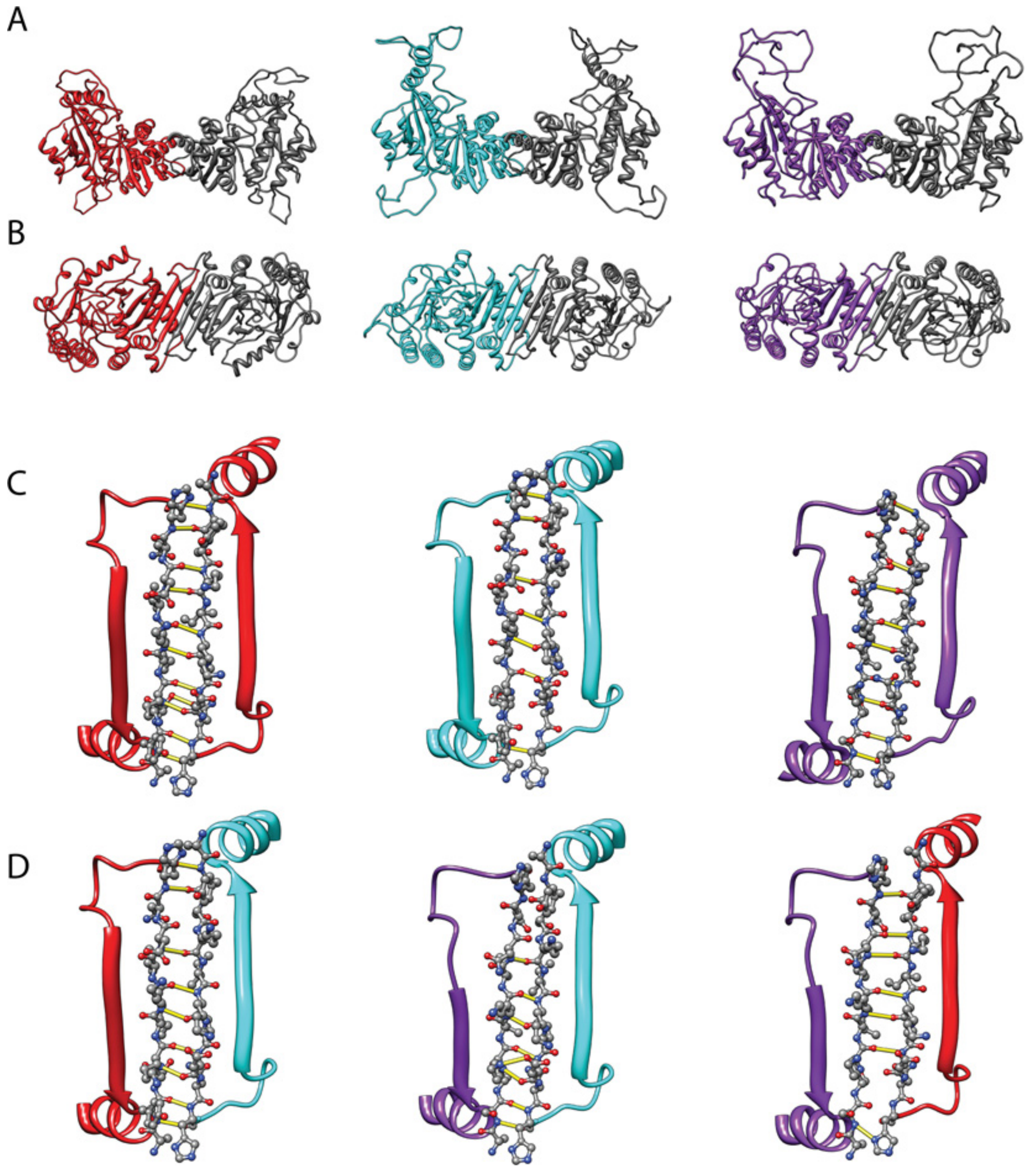

Figure S3 Predicted full structures of PI5P4Ks

(**A** and **B**) Amino-acid sequence and X-ray crystallography structures of all three isoforms were used to model the unordered regions and suggest possible conformations. The structures are presented as homodimers with one monomer coloured (PIP4Kα, red; PIP4Kβ, cyan; and PIP4Kγ, purple) and dimers are positioned to view the side elevation (**A**) and the flat surface that would be in contact with a membrane (**B**). (**C** and **D**) Dimerization interfaces for PI5P4K homo- and hetero-dimers. Fragments shown represent the β1 sheet interface (molecular view) attached to the β2 sheet region (ribbon view), with predicted hydrogen bonding shown in yellow. Bonding across the homodimer interfaces is predicted from the published structures (**C**) and predictions of heterodimerization interfaces were generated by superimposing the structure for the first 120 amino acids of an isoform on to one chain of the homodimer crystal structure of the pairing isoform and identifying the comparative hydrogen bond lengths using the FindHBond function of Chimera (**D**).

© 2013 The Author(s)
The author(s) has paid for this article to be freely available under the terms of the Creative Commons Attribution Licence (CC-BY) (http://creativecommons.org/licenses/by/3.0/) which permits unrestricted use, distribution and reproduction in any medium, provided the original work is properly cited.

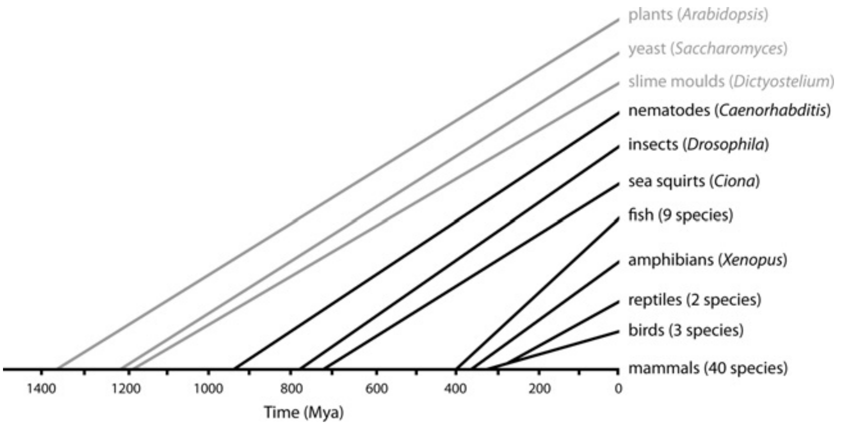

Figure S4 Evolutionary divergence of species with annotated PI4P5K/PI5P4K superfamily genes, relative to mammalian evolution

Groups denote taxa with only PI4P5K activity (light shading) and taxa with at least one PI5P4K activity (dark shading), as defined by gene sequence homology. The presence of genes in other lower metazoa (not annotated) has not been included (see Table S4). Timescale represents millions of years (since present). Values for estimated divergence of taxa expressing PI5P4K activity were estimated using TimeTree [2].

Table S1 Example turnover numbers from the BRENDA enzyme database

The examples show the maximum and minimum range of all known enzymes and also specifically of kinases. The k_{cat} values reported are for wild-type human enzymes utilizing natural substrates.

Enzyme	k_{cat} (s^{-1})
Histone-arginine N-methyltransferase	2.0×10^{-6}
Neutrophil collagenase	2.3×10^{-4}
Calpain-1	2.0×10^{-2}
Alcohol dehydrogenase	2.5
Deoxyribonuclease I	703
Cholinesterase	1433
Carbonic anhydratase II	1.4×10^{6}
Ethanolamine kinase	1.6×10^{-4}
Polo kinase	9.4×10^{-3}
Thiamine diphosphokinase	6.9×10^{-2}
Mitogen-activated protein kinase	0.9
Diacylglycerol kinase	1.7
Ins(1,3,4)P_3 5/6-kinase	20
Diphosphoinositol-pentakisphosphate kinase	38
Choline kinase	71.5
Creatine kinase	143
Adenylate kinase	842
Phosphoglycerate kinase	2633

Table S2 Regions of amino acids relating to structural variations in aligned models

Region 1 is synonymous with the putative G-loop sequence, region 2 with the variable insert sequence and region 3 with the activation loop, as designated in the PI5P4Kβ structure [1].

Region	PI5P4Kα	PI5P4Kβ	PI5P4Kγ
Region 1	125–133	130–138	133–140
Region 2	284–332	289–342	291–347
Region 3	363–387	372–397	378–402

Table S3 Evidence for PI5P4K activity in metazoan genomes

A BLAST search of the EnsemblMetazoa genomes using the dPIP4K peptide sequence from *D. melanogaster* (bold) matched regions of genomes from other species, suggesting that at least one similar sequence is present. Using *D. melanogaster* as a search control, the best percentage identity scores are reported over the largest alignment length. Species below the parasitoid wasp have ambiguity over the presence of a potential PI5P4K activity. The presence of regions of translated protein identity does not confirm the presence of a functional activity in these species. BLAST searches of the EnsemblPlants, EnsemblFungi, EnsemblProtists and EnsemblBacteria genome databases did not produce any matches greater than 35%, 40%, 34% or 29% identity respectively.

Common name	Latin name	Identity (%)	Length of alignment
**Fruitfly**	***Drosophila melanogaster***	**100**	**404**
Red flour beetle	*Tribolium castaneum*	72	415
Human louse	*Pediculus humanus*	73	394
Waterflea	*Daphnia pulex*	65	331
European honeybee	*Apis mellifera*	65	307
Nematode	*Trichinella spiralis*	50	321
Monarch butterfly	*Danaus plexippus*	68	298
Silkmoth	*Bombyx mori*	62	299
Pea aphid	*Acyrthosiphon pisum*	60	423
Trichoplax	*Trichoplax adhaerens*	47	404
Trematode	*Schistosoma mansoni*	49	402
Nematode	*Caenorhabditis elegans*	53	411
Deer tick	*Ixodes scapularis*	58	372
Starlet sea anenome	*Nematostella vectensis*	57	319
Purple sea urchin	*Strongylocentrotus purpuratus*	46	337
Leafcutter ant	*Atta cephalotes*	58	247
Nematode	*Pristionchus pacificus*	61	104
Parasitoid wasp	*Nasonia vitripennis*	96	33
Southern house mosquito	*Culex quinquefasciatus*	31	279
Yellow fever mosquito	*Aedes aegypti*	31	279
Sponge	*Amphimedon queenslandica*	40	205
Mosquito	*Anopheles gambiae*	28	214
Postman butterfly	*Heliconius melpomene*	36	197

© 2013 The Author(s)
The author(s) has paid for this article to be freely available under the terms of the Creative Commons Attribution Licence (CC-BY) (http://creativecommons.org/licenses/by/3.0/) which permits unrestricted use, distribution and reproduction in any medium, provided the original work is properly cited.

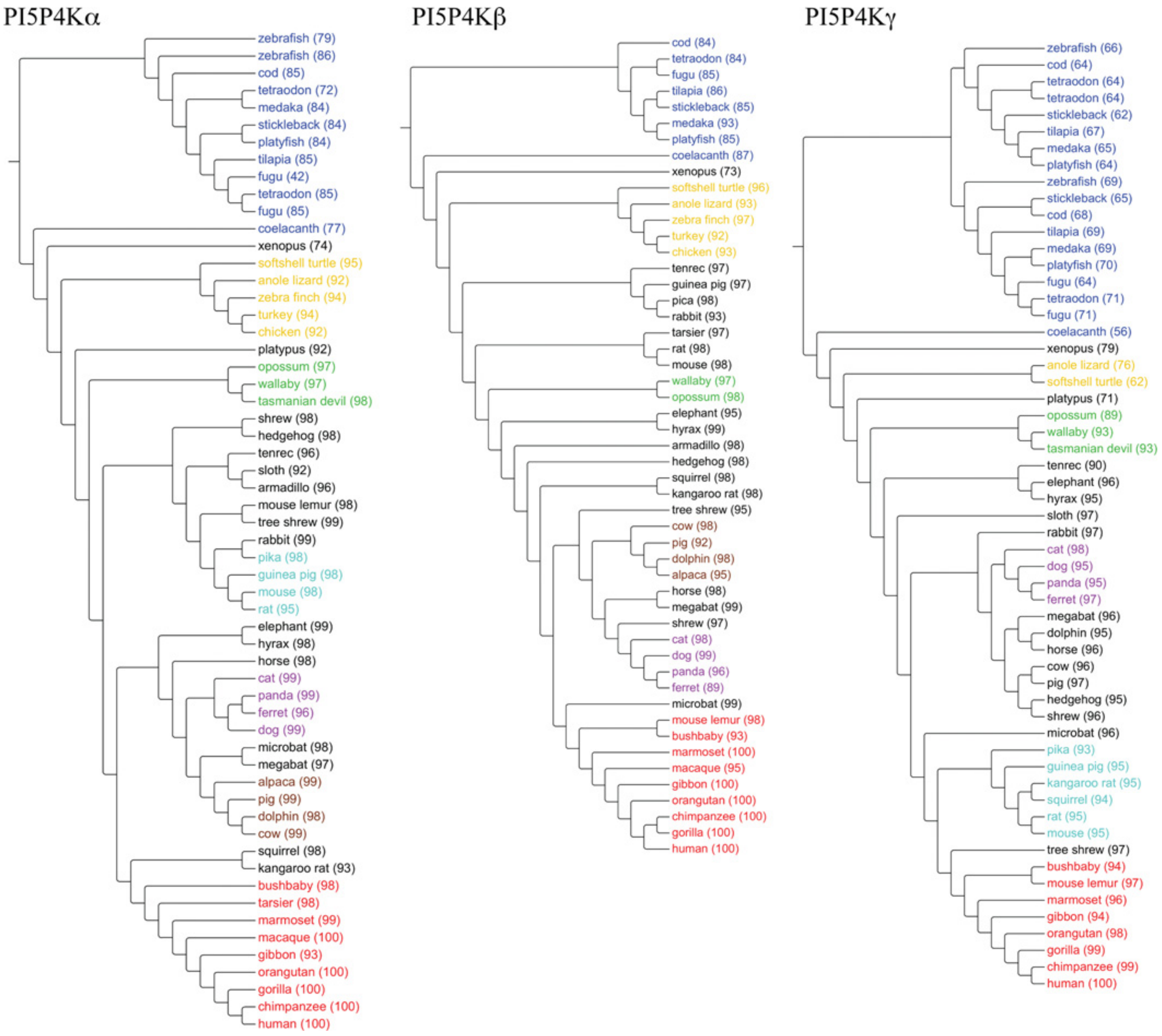

Figure S5 Molecular evolution of PI5P4K isoforms

Cladograms were created from phylograms (generated using the EnsemblCompara GeneTrees resource) to depict evolutionary divergence of PI5P4Ks in sequenced genomes. Numbers in parentheses represent percentage identity between orthologues. Scores were calculated from ClustalW pairwise alignments (using the BLOSUM sequence identity matrix) over regions of (partial) sequence available [3]. Coloured groups indicate related taxa (red, primates; brown, cetaceans and ungulates; purple, carnivores; cyan, rodents; green, marsupials; yellow, reptiles and birds; blue, fish).

© 2013 The Author(s)
The author(s) has paid for this article to be freely available under the terms of the Creative Commons Attribution Licence (CC-BY) (http://creativecommons.org/licenses/by/3.0/) which permits unrestricted use, distribution and reproduction in any medium, provided the original work is properly cited.

Table S4 Annotated PI5P4K genes in vertebrate genomes

The search was limited to complete sequenced genomes available on the Ensembl (EMBL-EBI) database using gene name and orthologue searches. The available genomes were current as of 1 January 2013.

PIP4K2A gene(s)	PIP4K2B gene(s)	PIP4K2C gene(s)	Common name	Latin name	Class, order
2	0	2	Zebrafish	*Danio rerio*	Actinopterygii, Cypriniformes
1	1	2	Platyfish	*Xiphophorus maculatus*	Actinopterygii, Cyprinodontiformes
1	1	2	Medaka (Japanese killifish)	*Oryzias latipes*	Actinopterygii, Beloniformes
1	1	2	Stickleback	*Gasterosteus aculeatus*	Actinopterygii, Gasterosteiformes
2	1	2	Pufferfish	*Takifugu rubripes*	Actinopterygii, Tetraodontiformes
2	1	3	Green-spotted pufferfish	*Tetraodon nigroviridis*	Actinopterygii, Tetraodontiformes
1	1	2	Tilapia	*Oreochromis niloticus*	Actinopterygii, Perciformes
1	1	2	Cod	*Gadus morhua*	Actinopterygii, Gadiformes
1	1	1	Coelacanth	*Latimeria chalumnae*	Sarcopterygii, Coelacanthiformes
1	1	1	Western clawed frog	*Xenopus tropicalis*	Amphibia, Anura
1	1	1	Chinese softshell turtle	*Pelodiscus sinensis*	Sauropsida, Testudines
1	1	1	Anole lizard	*Anolis carolinensis*	Reptilia, Squamata
1	1	0	Turkey	*Meleagris gallopavo*	Aves, Galliformes
1	1	0	Chicken	*Gallus gallus*	Aves, Galliformes
1	1	0	Zebra finch	*Taeniopygia guttata*	Aves, Passeriformes
1	1	1	Platypus	*Ornithorhynchus anatinus*	Mammalia, Monotremata
1	0	1	Tasmanian devil	*Sarcophilus harrisii*	Mammalia, Marsupialia
1	1	1	Wallaby	*Macropus eugenii*	Mammalia, Marsupialia
1	1	1	Opossum	*Monodelphis domestica*	Mammalia, Didelphimorphia
1	0	1	Sloth	*Choloepus hoffmanni*	Mammalia, Pilosa
1	1	0	Armadillo	*Dasypus novemcinctus*	Mammalia, Cingulata
1	1	1	Lesser hedgehog tenrec	*Echinops telfairi*	Mammalia, Afrosoricida
1	1	1	Hyrax	*Procavia capensis*	Mammalia, Hyracoidea
1	1	1	Elephant	*Loxodonta africana*	Mammalia, Proboscidea
1	1	1	Shrew	*Sorex araneus*	Mammalia, Soricomorpha
1	1	1	Hedgehog	*Erinaceus europaeus*	Mammalia, Erinaceomorpha
1	1	1	Megabat	*Pteropus vampyrus*	Mammalia, Chiroptera
1	1	1	Microbat	*Myotis lucifugus*	Mammalia, Chiroptera
1	1	1	Ferret	*Mustela putorius furo*	Mammalia, Carnivora
1	1	1	Dog	*Canis familiaris*	Mammalia, Carnivora
1	1	1	Panda	*Ailuropoda melanoleuca*	Mammalia, Carnivora
1	1	1	Cat	*Felis catus*	Mammalia, Carnivora
1	1	1	Horse	*Equus caballus*	Mammalia, Perissodactyla
1	1	1	Pig	*Sus scrofa*	Mammalia, Artiodactyla
1	1	1	Cow	*Bos taurus*	Mammalia, Artiodactyla
1	1	0	Alpaca	*Vicugna pacos*	Mammalia, Artiodactyla
1	1	1	Dolphin	*Tursiops truncatus*	Mammalia, Cetacea
1	1	1	Pika	*Ochotona princeps*	Mammalia, Lagomorpha
1	1	1	Rabbit	*Oryctolagus cuniculus*	Mammalia, Lagomorpha
1	1	1	Ground squirrel	*Ictidomys tridecemlineatus*	Mammalia, Rodentia
1	1	1	Guinea pig	*Cavia porcellus*	Mammalia, Rodentia
1	1	1	Kangaroo rat	*Dipodomys ordii*	Mammalia, Rodentia
1	1	1	Rat	*Rattus norvegicus*	Mammalia, Rodentia
1	1	1	Mouse	*Mus musculus*	Mammalia, Rodentia
1	1	1	Tree shrew	*Tupaia belangeri*	Mammalia, Scandentia
1	1	1	Bushbaby	*Otolemur garnettii*	Mammalia, Primates
1	1	1	Mouse lemur	*Microcebus murinus*	Mammalia, Primates
1	1	0	Tarsier	*Tarsius syrichta*	Mammalia, Primates
1	1	1	Marmoset	*Callithrix jacchus*	Mammalia, Primates
1	1	1	Macaque	*Macaca mulatta*	Mammalia, Primates
1	1	1	Gibbon	*Nomascus leucogenys*	Mammalia, Primates
1	1	1	Orangutan	*Pongo abelii*	Mammalia, Primates
1	1	1	Gorilla	*Gorilla gorilla*	Mammalia, Primates
1	1	1	Chimpanzee	*Pan troglodytes*	Mammalia, Primates
1	1	1	Human	*Homo sapiens*	Mammalia, Primates

© 2013 The Author(s)
The author(s) has paid for this article to be freely available under the terms of the Creative Commons Attribution Licence (CC-BY) (http://creativecommons.org/licenses/by/3.0/) which permits unrestricted use, distribution and reproduction in any medium, provided the original work is properly cited.

PI5P4Kα (281-305)	HDVERAEQEEVECEENDGEEEGESD
Prediction: GOR V	CCHHHHHHHHHHHCCCCCCCCCCCC
Prediction: PSIPRED [confidence]	CHHHHHHHHHHHHHHCCCCCCCCCC<br>9566765544212104664225579

PI5P4Kβ (286-310)	HDVDRAEQEEMEVEERAEDEECEND
Prediction: GOR V	CCCCHHHHHHHHHHHHHHHHHHCCC
Prediction: PSIPRED [confidence]	CHHHHHHHHHHHHHHHHHHHHHHCC<br>9036899899999887666766509

PI5P4Kγ (288-312)	HDIIRGSEPEEEAPVREDESEVDGD
Prediction: GOR V	CCCCCCCCCCCCCCCCCCCCCCCCC
Prediction: PSIPRED [confidence]	CCCCCCCCCCCCCCCCCCCCCCCCC<br>9643589866458863356422489

Figure S6 Prediction of secondary structure for the NLS regions of PI5P4K isoforms

Amino acid numbers are given in parentheses. Secondary structure prediction was performed by two separate methods, GOR V and PSIPRED v3.2 [4,5] with reported prediction accuracies (Q_3) of 73.5% and 81.6% respectively. Prediction lettering follows the Dictionary of Protein Secondary Structure (DSSP) format; H, 4-turn helix (α-helix); C, coil. Confidence is an arbitrary value 0–9 for the PSIPRED prediction.

REFERENCES

1 Rao, V. D., Misra, S., Boronenkov, I. V., Anderson, R. A. and Hurley, J. H. (1998) Structure of type IIβ phosphatidylinositol phosphate kinase: a protein kinase fold flattened for interfacial phosphorylation. Cell **94**, 829–839

2 Hedges, S. B., Dudley, J. and Kumar, S. (2006) TimeTree: a public knowledge-base of divergence times among organisms. Bioinformatics **22**, 2971–2972

3 Larkin, M. A., Blackshields, G., Brown, N. P., Chenna, R., McGettigan, P. A., McWilliam, H., Valentin, F., Wallace, I. M., Wilm, A., Lopez, R. et al. (2007) Clustal W and Clustal X version 2.0. Bioinformatics **23**, 2947–2948

4 Buchan, D. W., Ward, S. M., Lobley, A. E., Nugent, T. C., Bryson, K. and Jones, D. T. (2010) Protein annotation and modelling servers at University College London. Nucleic Acids Res. **38**, W563–W568

5 Jones, D. T. (1999) Protein secondary structure prediction based on position-specific scoring matrices. J. Mol. Biol. **292**, 195–202

Received 8 April 2013/7 June 2013; accepted 12 June 2013
Published as BJ Immediate Publication 12 June 2013, doi:10.1042/BJ20130488

© 2013 The Author(s)
The author(s) has paid for this article to be freely available under the terms of the Creative Commons Attribution Licence (CC-BY) (http://creativecommons.org/licenses/by/3.0/) which permits unrestricted use, distribution and reproduction in any medium, provided the original work is properly cited.
